# Analysis of macrophage polarization and regulation characteristics in ovarian tissues of polycystic ovary syndrome

**DOI:** 10.3389/fmed.2024.1417983

**Published:** 2024-09-11

**Authors:** Yue Yuan, Yan Mao, Liu Yang, Yilin Wang, Xuehong Zhang

**Affiliations:** ^1^The First Hospital of Lanzhou University, Lanzhou, China; ^2^The First School of Clinical Medicine, Lanzhou University, Lanzhou, China; ^3^Key Laboratory for Reproductive Medicine and Embryo of Gansu, Lanzhou, China; ^4^Gansu Provincial Hosipital, Lanzhou, China

**Keywords:** polycystic ovary syndrome, macrophage polarization, immune microenvironment, differentially expressed genes, macrophage polarization-related genes

## Abstract

**Background:**

Polycystic ovary syndrome (PCOS) can lead to infertility and increase the risk of endometrial cancer. Analyzing the macrophage polarization characteristics in ovarian tissues of PCOS is crucial for clinical treatment.

**Methods:**

We obtained 13 PCOS and nine control ovarian samples from the CEO database and analyzed differentially expressed genes (DEGs). Macrophage polarization-related genes (MPRGs) were sourced from the GeneCards and MSigDB databases. Intersection of DEGs with MPRGs identified DEGs associated with macrophage polarization (MPRDEGs). Gene ontology (GO), Kyoto Encyclopedia of Genes and Genomes (KEGG), and Protein–protein interaction (PPI) Network analysis were conducted on MPRDEGs. Moreover, the top 10 genes from three algorithms were identified as the hub genes of MPRGs. In addition, miRNAs, transcription factors (TFs), and drugs were retrieved from relevant databases for regulatory network analysis of mRNA-miRNA, mRNA-TF, and mRNA-Drug interactions. Immune cell composition analysis between the PCOS and control groups was performed using the CIBERSORT algorithm to calculate correlations across 22 immune cell types.

**Results:**

A total of 13 PCOS samples and nine control ovarian samples were obtained in this study. We identified 714 DEGs between the two groups, with 394 up-regulated and 320 down-regulated. Additionally, we identified 774 MPRGs, from which we derived 30 MPRDEGs by intersecting with DEGs, among which 21 exhibited interaction relationships. GO and KEGG analyses revealed the enrichment of MPRDEGs in five biological processes, five cell components, five molecular functions, and three biological pathways. Immune infiltration analysis indicated a strong positive correlation between activated nature killer (NK) cells and memory B cells, while neutrophils and monocytes showed the strongest negative correlation. Further investigation of MPRDEGs identified nine hub genes associated with 41 TFs, 82 miRNAs, and 44 drugs or molecular compounds. Additionally, qRT-PCR results demonstrated overexpression of the CD163, TREM1, and TREM2 genes in ovarian tissues from the PCOS group.

**Conclusion:**

This study elucidated the polarization status and regulatory characteristics of macrophages in ovarian tissues of the PCOS subjects, confirming significant overexpression of CD163, TREM1, and TREM2. These findings contribute to a deeper understanding of the pathogenesis of PCOS.

## 1 Introduction

Polycystic ovary syndrome (PCOS) is a prevalent reproductive and metabolic disorder affecting women of childbearing age. It is associated with a range of symptoms, including menstrual irregularities, hyperandrogenism, and even fertility failure (1, 2). Furthermore, multiple cohort studies indicate that PCOS patients have significantly higher rates of hypertension, renal disease, gastrointestinal disorders, eating disorders, mental health issues, depression, anxiety, rheumatoid arthritis, respiratory infections, obesity, dyslipidemia, non-alcoholic fatty liver disease, type 2 diabetes, cardiovascular diseases, dementia, and endometrial cancer compared to controls (3, 4), this underscores the critical importance of early prevention and detection of PCOS. According to WHO reports, approximately 8–20% of women worldwide suffer from PCOS, and effective treatment options are currently limited (1, 5, 6). A thorough analysis of the immunopathological characteristics of PCOS-related diseases will contribute to advancing global women’s healthcare.

Recent research highlights that dysregulation of macrophage polarization within the immune microenvironment of ovarian tissue may contribute significantly to ovulatory dysfunction and impaired egg maturation in PCOS (7–10), although the exact mechanisms remain unclear. Therefore, a detailed exploration of macrophage polarization characteristics within the local immune microenvironment of PCOS ovaries is essential for better understanding PCOS pathogenesis and advancing innovations in clinical interventions and preventive strategies. The ovarian immune microenvironment comprises diverse innate immune cells (such as macrophages, dendritic cells, and NK cells) and adaptive immune cells (including B and T cells), collectively maintaining normal ovarian function (8, 11). Alterations in the number and function of immune cells within this microenvironment can lead to compromised egg quality, impaired maturation, and ovulation disorders. Among these cells, macrophages are predominant in ovarian tissue and play a crucial role in ovarian function maintenance (12). Clinical evidence shows significant macrophage infiltration in the ovarian tissue of PCOS patients (10, 13), with a notably higher ratio of M1 to M2 macrophage compared to healthy individuals, a pattern also observed in PCOS animal models (14, 15).

Macrophage polarization is influenced by various factors, including local immune microenvironment, gene expression levels, and regulation by noncoding RNAs (ncRNAs) (16–19). In the early stages of PCOS, the immune microenvironment predominantly activates macrophages towards the M1 phenotype, leading to the release of proinflammatory cytokines such as interleukin-1 (IL-1), interleukin-6 (IL-6), interleukin-12 (IL-12), interferon-gamma (IFN-γ), and tumor necrosis factor-alpha (TNF-α), thereby initiating chronic low-grade inflammation (20–22). IL-1α inhibits plasminogen activator activity and interferes with regular ovulation, and IL-1β high expression harms oocyte maturation and fertilization rate (22, 23). IL-6 may cause ovarian dysfunction by interfering with follicle maturation (24); TNF-α reduces the production of progesterone and affects ovulation by inhibiting the expression of genes related to progesterone production (25, 26). In addition, M1 macrophages produce reactive oxygen species (ROS) that stimulate nitric oxide (NO) synthesis, exacerbating tissue damage (27). As PCOS progresses, macrophages can transition to the M2 phenotype under certain conditions, marked by overexpressing of surface receptors like a receptor for advanced glycation end-products (RAGE), a cluster of differentiation (CD) 163 (CD163), and CD206. M2 macrophages secrete C-C motif chemokine ligands (CCL) such as CCL24, CCL22, CCL17, and CCL18 (28, 29), which recruit eosinophils, basophils, and type 2 T helper (Th2) cells to the immune microenvironment (30, 31). M2 macrophages exhibit anti-inflammatory and immunomodulatory effects by promoting interleukin-10 (IL-10) and transforming growth factor-beta (TGF-β) production, which is crucial for maintaining local immune homeostasis in the ovaries (21). In PCOS patients, serum and follicular fluid levels of IL-1β, IL-6, IL-12, IL-23, and TNF-α are significantly elevated (32, 33), whereas IL-10 and TGF-β levels are reduced compared to the healthy individuals (34, 35). Triggering receptors expressed on myeloid cells (TREM) and Plexin-D1 (PLXND1) are implicated in promoting M1 macrophage polarization through inflammatory signal amplification and hemodynamic changes, respectively (36, 37). At the ncRNA level, microRNAs (miRNAs) play regulatory roles during macrophage polarization: miR-147 (38), miR-127 (39), miR-155 (40), miR-27a-3p (41), miR-30d-5p (42) and miRNA-19b-3p (43) are up-regulated in M1 macrophages, while miR-125a-5p (44), miR-143-3p (45), miR-145-5p (46), and miR-146a-3p (47) are up-regulated in M2 macrophages. miR379 inhibits M2 macrophage polarization, increasing the M1/M2 ratio and potentially affecting follicular development and ovulation in PCOS (48). The inflammatory response mediated by macrophage polarization significantly contributes to PCOS pathogenesis. Therefore, balancing the ratio of M1 to M2 macrophages may be crucial for improving PCOS outcomes (49).

However, the polarization characteristics, differential gene expression, and biological functions of macrophages in PCOS, particularly their involvement with miRNAs and signaling pathways, remain poorly understood. Therefore, this study aims to analyze differential gene expression related to macrophage polarization in PCOS tissues using a database approach. It will explore enriched signaling pathways and regulatory networks while thoroughly investigating immune cell infiltration patterns associated with PCOS. The focus will be on elucidating the differential expression and biological roles of genes related to macrophage polarization in PCOS, uncovering potential gene regulatory networks and drug targets. These findings aim to provide a theoretical basis for the clinical development of novel therapies targeting PCOS.

## 2 Materials and methods

### 2.1 Data download

The PCOS-related datasets GSE5850 (50) and GSE34526 (51) were retrieved from the Gene Expression Omnibus (GEO) database using the R package GEOquery (52). In our study, we analyzed the GSE5850 dataset, which contains oocytes at the metaphase II (MII) stage, and the GSE34526 dataset, which includes granulosa cells. Although these tissues are not traditionally classified as immune-related, research has suggested their interactions with the immune system within the ovarian microenvironment are of interest (10, 53). Furthermore, Liu L et al. have used these same datasets (GSE5850 and GSE34526) to investigate the role of chronic low-grade inflammation in the pathogenesis of PCOS (54). Their study, through functional enrichment analysis of immune cell infiltration, identified new diagnostic markers and potential small-molecule drugs, underscoring the importance of studying inflammatory responses in PCOS. Thus, our research employs the GSE5850 and GSE34526 datasets for related analytical investigations.

All samples originated from *Homo sapiens*. The chip platform utilized was GPL570, with detailed specifications provided in Table 1. Dataset GSE5850 comprises 6 PCOS samples and 6 control samples, while the dataset GSE34526 shall consist of 7 PCOS samples and 3 control samples. This study included all PCOS and control samples in these datasets.

**Table 1:** GEO microarray chip information.

	GSE5850	GSE34526
Platform	GPL570	GPL570
Species	*Homo sapiens*	*Homo sapiens*
Tissue	MII Arrested Oocyte	Granulosa Cell
Samples in PCOS Group	6	7
Samples in the Control Group	6	3
Reference	PMID: 17148555	PMID: 22904171

PCOS, Polycystic Ovary Syndrome; GEO, Gene Expression Omnibus.

Additionally, macrophage polarization-related genes (MPRGs) were gathered from the GeneCards database (55) and the Molecular Signatures Database (MSigDB) (56). GeneCards offers comprehensive information on human genes, where we utilized “Macrophage Polarization” as the search term, focusing specifically on “Protein Coding” genes, resulting in 623 MPRGs. Similarly, in MSigDB, using the keyword “Macrophage Polarization,” we identified 83 MPRGs from the COATES MACROPHAGE M1 *VS* M2 DN gene set and 86 MPRGs from the COATES MACROPHAGE M1 *VS* M2 UP gene set. After merging and removing duplicates, we obtained a total of 774 MPRGs. Detailed information is provided in Supplementary Table S1.

Furthermore, batch correction was performed on datasets GSE5850 and GSE34526 using the R package sva (57), integration a combined GEO dataset (Combined Datasets) comprising 13 samples from individuals with PCOS and nine control samples. Subsequently, the combined datasets were standardized and normalized, and probe annotations were conducted using the R package limma (58).

### 2.2 DEGs associated with macrophage polarization in PCOS

The R package limma was employed to conduct differential gene expression analysis comparing the PCOS and control groups. Differential expression was defined using thresholds of |log fold change (FC)| > 1 and *p*-value <0.05. Genes exhibiting logFC >1 and *p*-value <0.05 were categorized as up-regulated DEGs, while those with logFC<−1 and *p*-value <0.05 were categorized as down-regulated DEGs. To identify MPRDEGs associated with PCOS, we intersected all DEGs meeting the criteria of |logFC| > 1 and *p*-value <0.05 from the integrated GEO dataset with MPRGs. The Venn diagram was utilized to visualize the overlap and identify MPRDEGs. The differential analysis results were visualized using a volcano plot generated by the R package ggplot2 and a heatmap generated by the R package heatmap.

### 2.3 GO and KEGG enrichment analysis

GO is a commonly used method for large-scale functional enrichment analysis, which includes Biological Process (BP), Molecular Function (MF), and Cell Component (CC) (59). KEGG is a widely used database that stores information on genomes, biological pathways, diseases, drugs, and more (60). The present study used the R package clusterProfiler to perform GO and KEGG enrichment analysis on MPRDEGs. The entry screening criteria were adjusted to a *p* (adj. *p*) value less than 0.05 and a false discovery rate (FDR) value less than 0.25, which were considered statistically significant. The adj. *p* correction method used was Benjamini-Hochberg (BH).

### 2.4 Gene set enrichment analysis

Gene set enrichment analysis (GSEA) assesses the distribution pattern of genes within a predefined gene set in a gene expression dataset sorted by their correlation with phenotypes, aiming to determine their association with the phenotype (61). In this study, genes from the combined datasets were categorized into PCOS and Control. GSEA was performed on all genes in the integrated GEO dataset using the R package clusterProfiler based on their logFC values. The parameters for GSEA included a seed value of 2020, 1,000 permutations, a minimum gene set size of 10, a maximum gene set size of 500, and *p*-values corrected using the BH method. Gene sets used in GSEA were retrieved from the c2.all.v7.5.1.symbols.gmt gene set in MSigDB. Criteria for significant enrichment were set at *p*-value <0.05 and FDR < 0.25.

### 2.5 PPI network and hub gene selection

The PPI Network involves proteins interacting through mutual interactions (62). The Search Tool for the Retrieval of Interacting Genes/Proteins (STRING) database is utilized to explore known and predicted protein interactions (63). In this study, the PPI network specific to MPRDEGs was constructed using the STRING database, with a minimum required interaction score set at 0.400 (medium confidence level). The network was visualized using Cytoscape software (64).

In addition, the top 10 hub genes related to macrophage polarization were identified using three algorithms within the cytoHubba plugin (65): Maximum Neighborhood Component (MNC), Degree, and Maximal Clique Centrality (MCC) (66). First, scores for MPRDEGs in the PPI network were computed and ranked. Subsequently, the intersection of the top 10 genes from each algorithm was determined, and a Venn diagram was generated to analyze the overlap. Genes appearing in all three algorithm results were considered hub genes associated with macrophage polarization.

### 2.6 Construction of regulatory networks

miRNAs play a crucial regulatory role in biological development and evolution by targeting multiple genes, and conversely, a single gene can be regulated by various miRNAs. To explore the association between hub genes involved in macrophage polarization and miRNAs, miRNAs associated with these hub genes were extracted from the TarBase database (67), and an mRNA-miRNA regulatory network was constructed and visualized using Cytoscape software.

TFs govern gene expression by interacting with target genes post-transcriptionally. TFs that regulate the hub genes related to macrophage polarization were identified using data from the ChIPBase database (68). Subsequently, an mRNA-TF regulatory network was constructed using Cytoscape software to illustrate these regulatory interactions.

The comparative toxicogenomics database (CTD) was employed to predict both direct and indirect drug targets of hub genes associated with macrophage polarization (69). This exploration aimed to elucidate the interactions between hub genes and drugs. Finally, the mRNA-drug regulatory network was visualized using Cytoscape software to depict these regulatory relationships comprehensively.

### 2.7 Immune infiltration analysis

CIBERSORT is a linear support vector regression-based method used to deconvolute of transcriptome expression matrices, estimating the composition and abundance of immune cells within heterogeneous cell populations (70). By applying the CIBERSORT algorithm alongside the LM22 feature gene matrix and filtering data with immune cell enrichment scores above zero, specific results of immune cell infiltration matrices can be derived. Subsequently, correlation heatmaps are generated using the R package heatmap to visualize integrated GEO datasets (combined datasets) and illustrate correlation analyses between LM22 immune cells and hub genes associated with macrophage polarization.

### 2.8 Differential expression analysis of hub genes related to macrophage polarization

We generated grouping comparison plots based on the expression levels of these hub genes to further investigate differential expression related to macrophage polarization in the PCOS and control groups of the integrated GEO datasets. The Receiver Operating Characteristic (ROC) curve is an analysis tool used to assess model performance, distinguish optimal from suboptimal models, or establish optimal thresholds within a model (71). The ROC curve provides a comprehensive measure of sensitivity and specificity for continuous variables, demonstrating how well the model discriminates between classes. Using the R package ROC, ROC curves were plotted for hub genes associated with macrophage polarization in the integrated GEO datasets of PCOS, and the Area Under the Curve (AUC) was calculated to evaluate the diagnostic performance of these hub genes’ expression levels for PCOS. AUC values range between 0.5 and 1, where a value closer to 1 indicates better diagnostic performance. AUC values below 0.7 suggest low accuracy, values between 0.7 and 0.9 indicate moderate accuracy, and values 0.9 indicate high accuracy.

### 2.9 Animals and PCOS modeling

Twelve female mice of specific pathogen-free (SPF) grade, aged 3 weeks, were randomly divided into two groups of six mice each. Mice in the PCOS group received daily injections of dehydroepiandrosterone (DHEA) at a dose of 6 mg per 100 g body weight, dissolved in sesame oil, for 21 consecutive days. Mice in the control group received an equivalent volume of sesame oil daily. All mice were purchased from the Animal Experimental Center of Lanzhou University and housed in the SPF standardized laboratory animal facility at the Medical Experimental Center of Lanzhou University. DHEA was purchased from Shanghai McLean Biochemical Technology Co., Ltd. The study protocol was approved ty the Ethics Committee of Lanzhou University.

### 2.10 Hematoxylin and eosin (HE) staining

We utilized HE staining to evaluate polycystic ovarian changes in the animal models. Each mouse was anesthetized with 0.2 mL of 3% Pentobarbital Sodium and euthanized by neck dislocation. Both ovaries were surgically collected, and after the removal of fatty tissue and the capsule under an anatomical microscope, they were preserved intact. The left ovaries from both groups were fixed in 4% paraformaldehyde. In contrast, the right ovaries were frozen at −80°C for subsequent quantitative real-time polymerase chain reaction (qRT-PCR) and further analyses. The fixed ovaries underwent routine processing, including dehydration, paraffin embedding, sectioning at 5 μM thickness, dewaxing, rehydration, and staining with HE according to standard protocols (72).

### 2.11 qRT-PCR analysis

The mRNA levels of DEGs including allograft inflammatory factor 1 (AIF1), CD163, TREM1, TREM2, granular protein (GRN), and heat shock protein family A member 5 (HSPA5) in ovarian tissue were quantified by qRT-PCR analysis. Total RNA extraction was performed with RNAkey™ Reagent (SEVEN, China), followed by reverse transcription using FastKing gDNA Dispelling RT SuperMix (KR118, TIANGEN Biotech). PCR primer sequences were designed using DNAMAN software, and the amplification protocol consisted of 40 cycles of denaturation at 95°C for 30 s and annealing/extension at 55°C for 20 s in a 20 μL reaction volume. Melting curve analysis included steps at 95°C, 60°C, and 95°C each for 15 s. GAPHD was employed as an internal reference gene. The qRT-PCR primer sequences used in this study are detailed in Table 2.

**Table 2:** The primer sequences used for qRT-PCR.

Name	Forward 5′ → 3′ primer	Reverse 5′ → 3′ primer
AIF1	GGATCTGCCGTCCAAAC	GCATTCGCTTCAAGGACA
CD163	ACATAGATCATGCATCTGTCATTTG	CATTCTCCTTGGAATCTCACTTCTA
TREM2	CCTCTCCACCAGTTTCTCCT	CAGTGCTTCAAGGCGTCATAAG
GRN	CTGTCGTGTGCCCTGATGCTAAG	CCCCAGTCCCCAGAATTGAGTTTG
HSPA5	TGTGGTACCCACCAAGAAGTC	TTCAGCTGTCACTCGGAGAAT
TREM1	ACTGCTGTGCGTGTTCTTTG	GCCTTCTGGCTGTTGGCATA
GAPDH	CGACTTCAACAGCAACTCCCACTCTTCC	TGGGTGGTCCAGGGTTTCTTACTCCTT

### 2.12 Statistical analysis

Data processing and analysis for this study were performed using R software (Version 4.2.0). Continuous variables are expressed as mean ± standard deviation. The Wilcoxon Rank Sum Test was applied to compare differences between two groups. Unless otherwise stated, Spearman correlation analysis was employed to determine the correlation coefficient between different molecules, with statistical significance set at *p*-value <0.05.

## 3 Results

### 3.1 Technology roadmap

**Figure 1:**
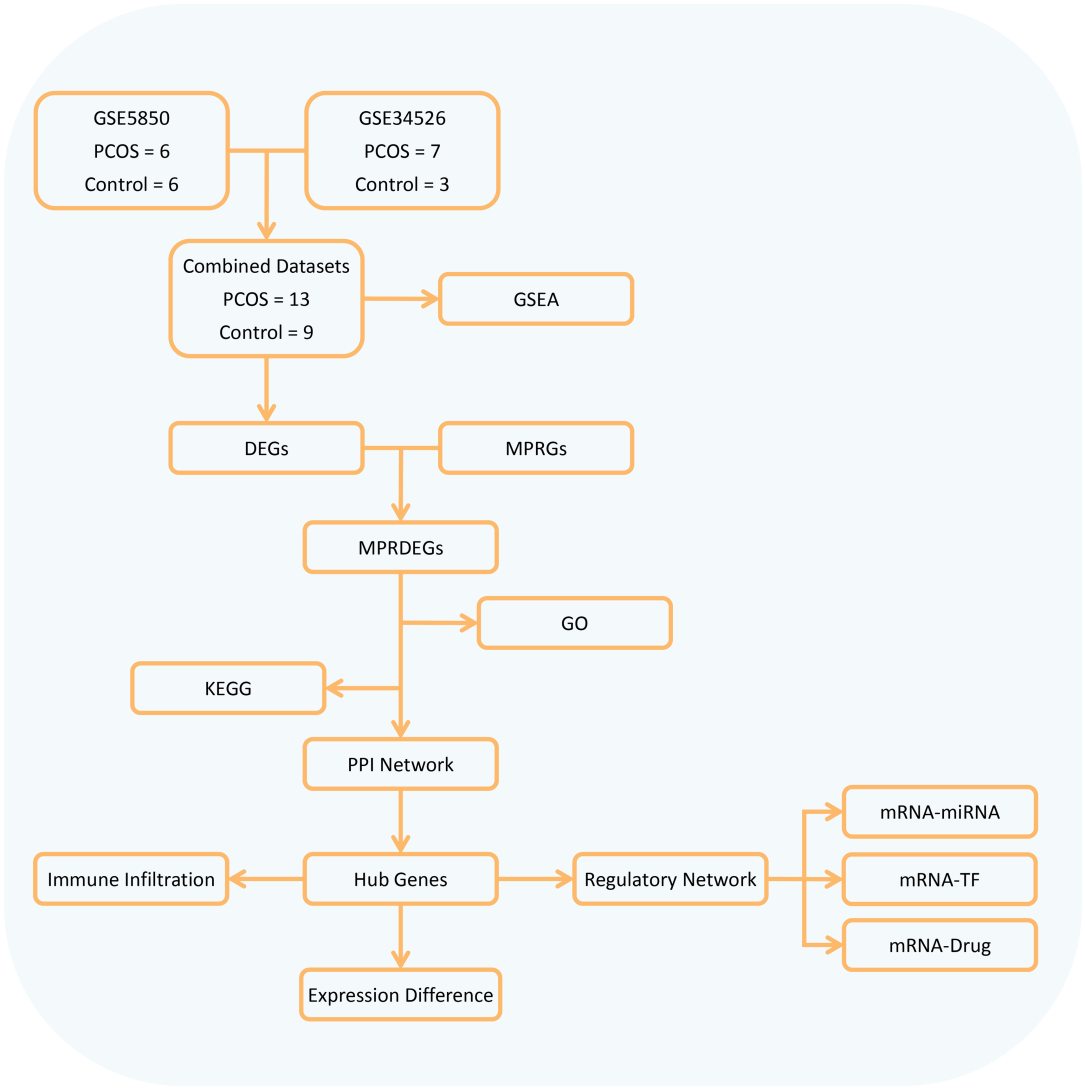
Flow chart for the comprehensive analysis of MPRDEGs. PCOS, Polycystic Ovary Syndrome; DEGs, Differentially Expressed Genes, MPRGs, Macrophage Polarization-Related Genes; MPRDEGs, Macrophage Polarization-Related Differentially Expressed Genes; GO, Gene Ontology; KEGG, Kyoto Encyclopedia of Genes and Genomes; GSEA, Gene Set Enrichment Analysis; PPI, Protein–protein Interaction; TF, Transcription Factor.

Our study obtained 13 PCOS and 9 control samples from two major databases. It conducted differential gene analysis between these groups and identified MPRDEGs through the intersection of DEGs and MPRGs. Subsequently, GO, KEGG, and PPI network analyses were performed on the MPRDEGs. Hub genes were identified from the PPI network, and their differential expression was validated. Additionally, the study included immune infiltration analysis and regulatory network analysis (mRNA-miRNA, mRNA-TF, and mRNA-Drug) of the hub genes. The overall research workflow is depicted in Figure 1.

### 3.2 Integration of polycystic ovary syndrome datasets

**Figure 2:**
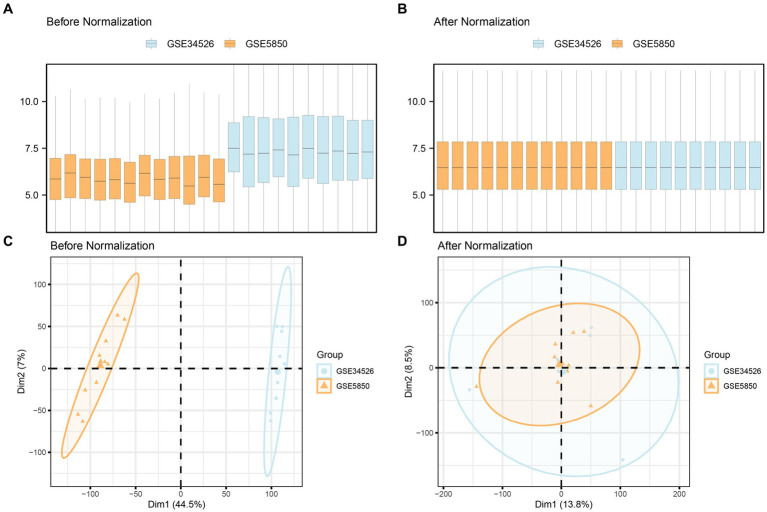
Batch effects removal of GSE5850 and GSE34526. **(A,B)** Boxplots of the combined datasets before **(A)** and after **(B)** batch effect removal. **(C,D)** PCA plots of the combined datasets before **(C)** and after **(D)** batch effect removal. PCA, Principal Component Analysis; PCOS, Polycystic Ovary Syndrome. The orange color represents the PCOS dataset GSE5850, and the light blue color represents the PCOS dataset GSE34526.

Initially, we combined datasets from the PCOS datasets GSE5850 and GSE34526 using the R package sva to mitigate batch effects. To assess the effectiveness of this process, we compared the datasets using distribution boxplots and Principal Component Analysis (PCA) plots (Figure 2). Both distribution boxplot and PCA plots indicate substantial reduction in batch effects among the PCOS dataset samples following batch effect removal.

### 3.3 Analysis of MPRDEGs in polycystic ovary syndrome

**Figure 3:**
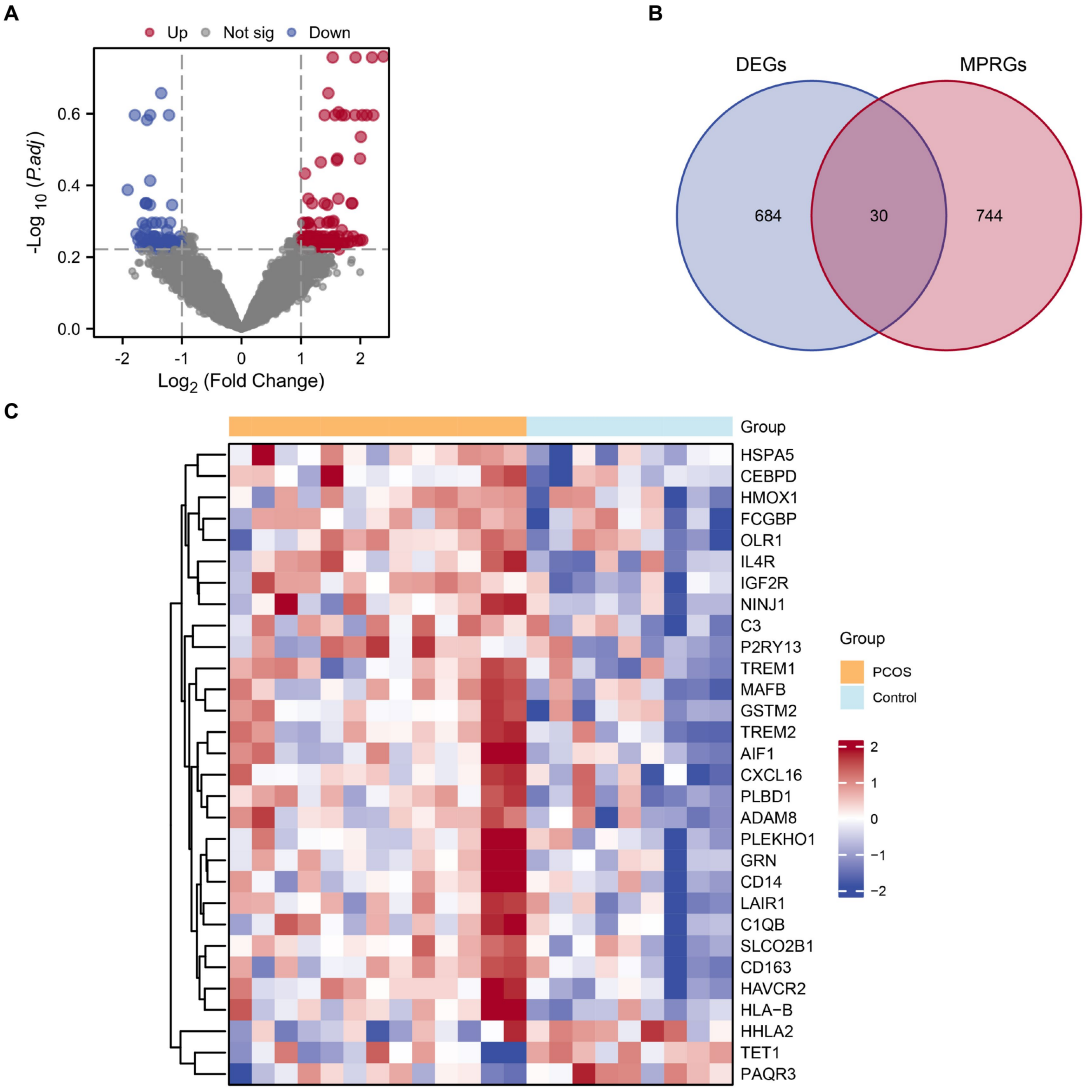
Combined datasets differential gene expression analysis. **(A)** Volcano plot of DEGs analysis between the PCOS and control groups in the combined datasets. **(B)** Venn diagram of DEGs and MPRGs in the combined datasets. **(C)** Heatmap of correlation of MPRDEGs in the combined datasets. PCOS, Polycystic Ovary Syndrome; DEGs, Differentially Expressed Genes; MPRGs, Macrophage Polarization-Related Genes; MPRDEGs, Macrophage Polarization-Related Differentially Expressed Genes. The light blue color represents the control group, and the orange color represents the PCOS group.

The combined datasets were stratified into PCOS and a control groups. Using the R package limma, we analyzed differential gene expression on the integrated GEO dataset, identifying 714 DEGs meeting the |logFC| > 1 criteria and *p*-value <0.05. Among them, 394 genes were up-regulated (logFC>1 and *p*-value <0.05), while 320 genes were down-regulated (logFC<−1 and *p*-value <0.05). A volcano plot visualizing there results is presented in Figure 3A. To identify MPRDEGs, we intersected all DEGs meeting the criteria |logFC| > 1 and *p* value <0.05 with MPRGs, resulting in 30 MPRDEGs, illustrated in a Venn diagram (Figure 3B). These MPRDEGs include IGF2R, HAVCR2, PLBD1, IL4R, TREM2, SLCO2B1, HSPA5, MAFB, GSTM2, LAIR1, HLA-B, ADAM8, GRN, CD163, NINJ1, C1QB, CXCL16, AIF1, TREM1, FCGBP, C3, CD14, CEBPD, OLR1, P2RY13, PLEKHO1, HMOX1, TET1, HHLA2, and PAQR3. Subsequently, we analyzed the expression differences of these MPRDEGs across different sample groups in the integrated GEO dataset and generated a heatmap using the R package heatmap to visualize the results (Figure 3C).

### 3.4 Enrichment analysis of GO and KEGG for MPRDEGs

**Figure 4:**
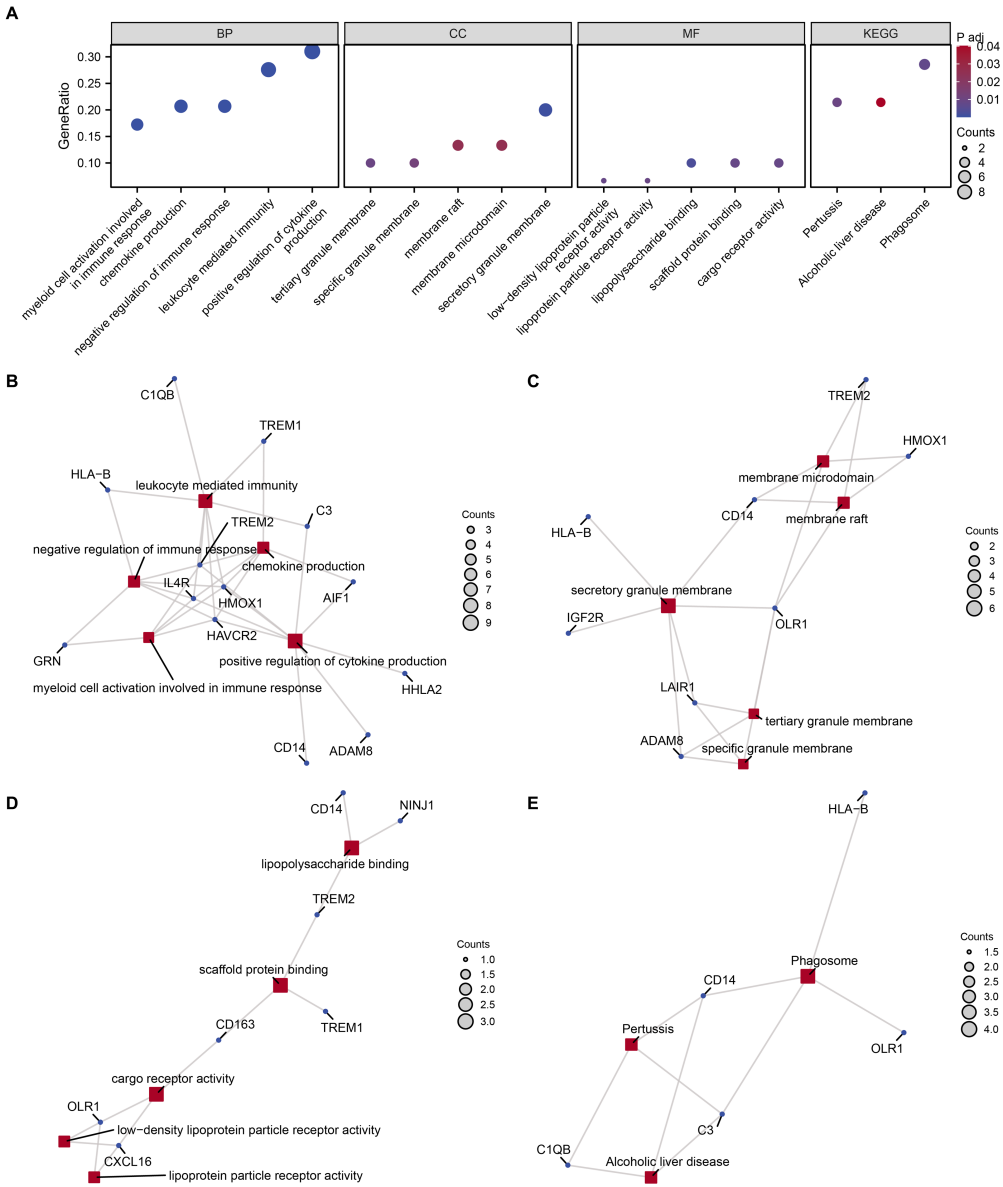
GO and KEGG enrichment analysis for MPRDEGs. **(A)** The bubble chart displays the results of GO and KEGG enrichment analysis for MPRDEGs: BP, CC, MF, and pathways. The horizontal axis shows GO terms and KEGG terms. **(B–E)** The network diagrams show the results of GO and KEGG enrichment analysis for MPRDEGs: BP **(B)**, CC **(C)**, MF **(D)**, and KEGG **(E)**. MPRDEGs, Macrophage Polarization-Related Differentially Expressed Genes; GO, Gene Ontology; KEGG, Kyoto Encyclopedia of Genes and Genomes; BP, Biological Process; CC, Cellular Component; MF, Molecular Function. The red nodes represent BP, CC, MF, and KEGG entries, while the dark blue nodes represent molecules. The connections define the relationships between entries and molecules. The screening criteria for GO and KEGG enrichment analysis were adj. *p* < 0.05 and an FDR value <0.25, which were considered statistically significant. The adj. *p* correction method used was the Benjamini-Hochberg method.

We conducted GO and KEGG enrichment analyses to explore the functional roles of the 30 identified MPRDEGs in PCOS. The results, presented in Table 3, reveal significant enrichments across several BP, MF, CC, and biological pathways. BP enrichments include chemokine production, positive regulation of cytokine production, negative regulation of immune response, myeloid cell activation involved in immune response, and leukocyte-mediated immunity in PCOS. CC enrichments encompass secretory granule membrane, tertiary granule membrane, specific granule membrane, membrane raft, and membrane microdomain. MF enrichments involve activities such as lipopolysaccharide binding, scaffold protein binding, low-density lipoprotein particle receptor activity, cargo receptor activity, and lipoprotein particle receptor activity. Additionally, MPRDEGs are involved in biological pathways, including Phagosome, Pertussis, and Alcoholic liver disease. The findings were visualized using a bubble chart (Figure 4A) to illustrate the significance of enrichments. Moreover, network diagrams (Figures 4B–E) were constructed based on the results of the GO and KEGG enrichment analyses, highlighting connections between molecules and annotated items. Nodes of greater significance indicate the involvement of more molecules in the respective item.

**Table 3:** Results of GO and KEGG enrichment analysis for MPRDEGs.

Ontology	ID	Description	GeneRatio	BgRatio	*p*-value	*p*. adjust	q-value
BP	GO:0032602	Chemokine production	6/29	98/18800	7.41E-09	1.01E-05	5.86E-06
BP	GO:0001819	Positive regulation of cytokine production	9/29	475/18800	2.49E-08	1.70E-05	9.83E-06
BP	GO:0050777	Negative regulation of immune response	6/29	179/18800	2.71E-07	8.08E-05	4.67E-05
BP	GO:0002275	Myeloid cell activation is involved in the immune response	5/29	93/18800	2.87E-07	8.08E-05	4.67E-05
BP	GO:0002443	Leukocyte mediated immunity	8/29	457/18800	3.14E-07	8.08E-05	4.67E-05
CC	GO:0030667	Secretory granule membrane	6/30	312/19594	6.68E-06	6.95E-04	4.64E-04
CC	GO:0070821	Tertiary granule membrane	3/30	73/19594	1.87E-04	9.74E-03	6.51E-03
CC	GO:0035579	Specific granule membrane	3/30	91/19594	3.59E-04	1.25E-02	8.32E-03
CC	GO:0045121	Membrane raft	4/30	326/19594	1.47E-03	2.57E-02	1.72E-02
CC	GO:0098857	Membrane microdomain	4/30	327/19594	1.48E-03	2.57E-02	1.72E-02
MF	GO:0001530	Lipopolysaccharide binding	3/30	34/18410	2.26E-05	2.71E-03	1.90E-03
MF	GO:0097110	Scaffold protein binding	3/30	67/18410	1.74E-04	8.52E-03	5.98E-03
MF	GO:0005041	Low-density lipoprotein particle receptor activity	2/30	15/18410	2.66E-04	8.52E-03	5.98E-03
MF	GO:0038024	Cargo receptor activity	3/30	79/18410	2.84E-04	8.52E-03	5.98E-03
MF	GO:0030228	Lipoprotein particle receptor activity	2/30	18/18410	3.86E-04	9.27E-03	6.51E-03
KEGG	hsa04145	Phagosome	4/14	152/8164	1.00E-04	7.40E-03	6.00E-03
KEGG	hsa05133	Pertussis	3/14	76/8164	2.62E-04	9.70E-03	7.86E-03
KEGG	hsa04936	Alcoholic liver disease	3/14	142/8164	1.63E-03	4.02E-02	3.26E-02
BP	GO:0032602	Chemokine production	6/29	98/18800	7.41E-09	1.01E-05	5.86E-06
BP	GO:0001819	Positive regulation of cytokine production	9/29	475/18800	2.49E-08	1.70E-05	9.83E-06

GO, Gene Ontology; BP, Biological Process; CC, Cellular Component; MF, Molecular Function; KEGG, Kyoto Encyclopedia of Genes and Genomes; MPRDEGs, Macrophage Polarization-Related Differentially Expressed Genes.

### 3.5 Gene set enrichment analysis

**Figure 5:**
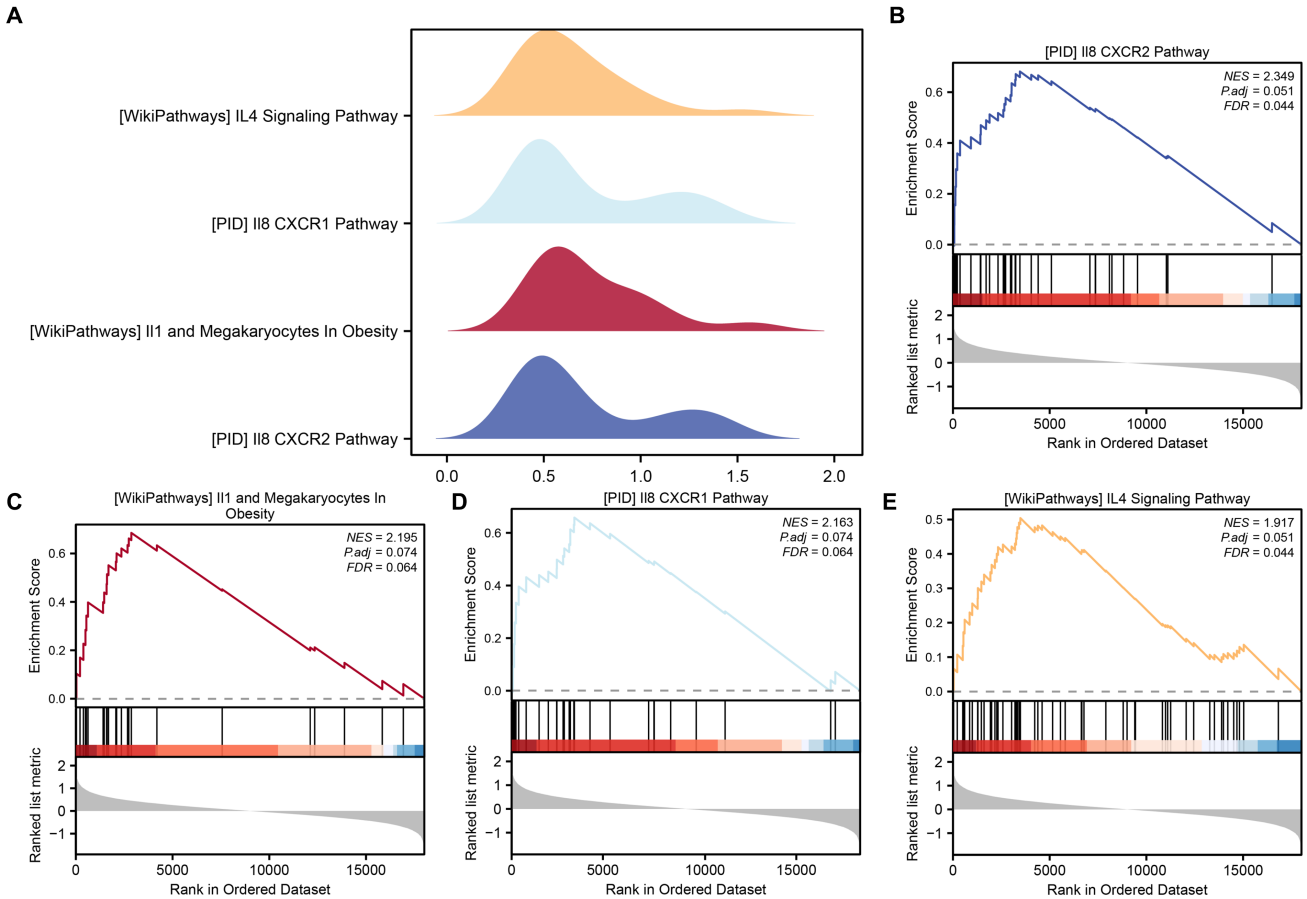
GSEA for combined datasets. **(A)** The GSEA of four biological functions in the combined GEO dataset is shown in mountain plots. **(B–E)** The GSEA showed that PCOS significantly affected PID_IL8_CXCR2_PATHWAY **(B)**, WP_IL1_AND_MEGAKARYOCYTES_IN_OBESITY **(C)**, PID_IL8_CXCR1_PATHWAY **(D)**, and WP_IL4_SIGNALING_PATHWAY **(E)**. The screening criteria for GSEA were *p* value <0.05 and FDR value <0.25.

To assess the collective impact of all gene expression levels from the combined GEO dataset on PCOS, we conducted GSEA to explore their involvement in biological processes, cellular components, and molecular functions (Figure 5A). The detailed outcomes are summarized in Table 4. The analysis revealed significant associations between gene expression and various biological functions and signaling pathways. Specifically, pathways such as PID_IL8_CXCR2_PATHWAY (Figure 5B), WP_IL1_ AND _MEGAKARYOCYTES _ IN_OBESITY (Figure 5C), _IL8_CXCR1_PATHWAY (Figure 5D), and WP_I L4_ SIGNALING_PATHWAY (Figure 5E) were prominently affected by the gene expression profiles in the integrated GEO dataset.

**Table 4:** Results of gene set enrichment analysis for combined datasets.

ID	setSize	Enrichment score	NES	*p*-value	*q*-values
WP_MICROGLIA_PATHOGEN_PHAGOCYTOSIS_PATHWAY	40	7.31E-01	2.64E+00	1.98E-03	4.38E-02
REACTOME_NEUTROPHIL_DEGRANULATION	448	5.08E-01	2.61E+00	1.65E-03	4.38E-02
WP_TYROBP_CAUSAL_NETWORK_IN_MICROGLIA	59	6.32E-01	2.46E+00	1.98E-03	4.38E-02
REACTOME_INTERFERON_ALPHA_BETA_SIGNALING	70	5.95E-01	2.41E+00	1.89E-03	4.38E-02
PID_IL8_CXCR2_PATHWAY	34	6.81E-01	2.35E+00	1.96E-03	4.38E-02
REACTOME_IMMUNOREGULATORY_INTERACTIONS_BETWEEN_A_LYMPHOID_AND_A_NON_LYMPHOID_CELL	124	5.28E-01	2.35E+00	1.81E-03	4.38E-02
KEGG_ANTIGEN_PROCESSING_AND_PRESENTATION	74	5.66E-01	2.31E+00	1.89E-03	4.38E-02
REACTOME_INTERLEUKIN_10_SIGNALING	43	6.26E-01	2.28E+00	2.01E-03	4.38E-02
KEGG_LEISHMANIA_INFECTION	67	5.67E-01	2.28E+00	1.93E-03	4.38E-02
KEGG_VIRAL_MYOCARDITIS	65	5.68E-01	2.26E+00	1.93E-03	4.38E-02
KEGG_ALLOGRAFT_REJECTION	33	6.56E-01	2.24E+00	1.96E-03	4.38E-02
REACTOME_PURINERGIC_SIGNALING_IN_LEISHMANIASIS_INFECTION	26	6.87E-01	2.22E+00	1.98E-03	4.38E-02
KEGG_SYSTEMIC_LUPUS_ERYTHEMATOSUS	50	5.90E-01	2.21E+00	2.00E-03	4.38E-02
REACTOME_GENERATION_OF_SECOND_MESSENGER_MOLECULES	29	6.57E-01	2.20E+00	1.96E-03	4.38E-02
WP_TYPE_II_INTERFERON_SIGNALING	36	6.28E-01	2.20E+00	1.94E-03	4.38E-02
WP_IL1_AND_MEGAKARYOCYTES_IN_OBESITY	24	6.85E-01	2.19E+00	3.92E-03	6.38E-02
KEGG_TYPE_I_DIABETES_MELLITUS	39	6.12E-01	2.19E+00	1.98E-03	4.38E-02
REACTOME_PD_1_SIGNALING	19	7.34E-01	2.18E+00	2.00E-03	4.38E-02
PID_IL8_CXCR1_PATHWAY	27	6.58E-01	2.16E+00	3.91E-03	6.38E-02
WP_IL4_SIGNALING_PATHWAY	54	5.04E-01	1.92E+00	2.00E-03	4.38E-02

### 3.6 Construction of PPI network and screening of hub genes

**Figure 6:**
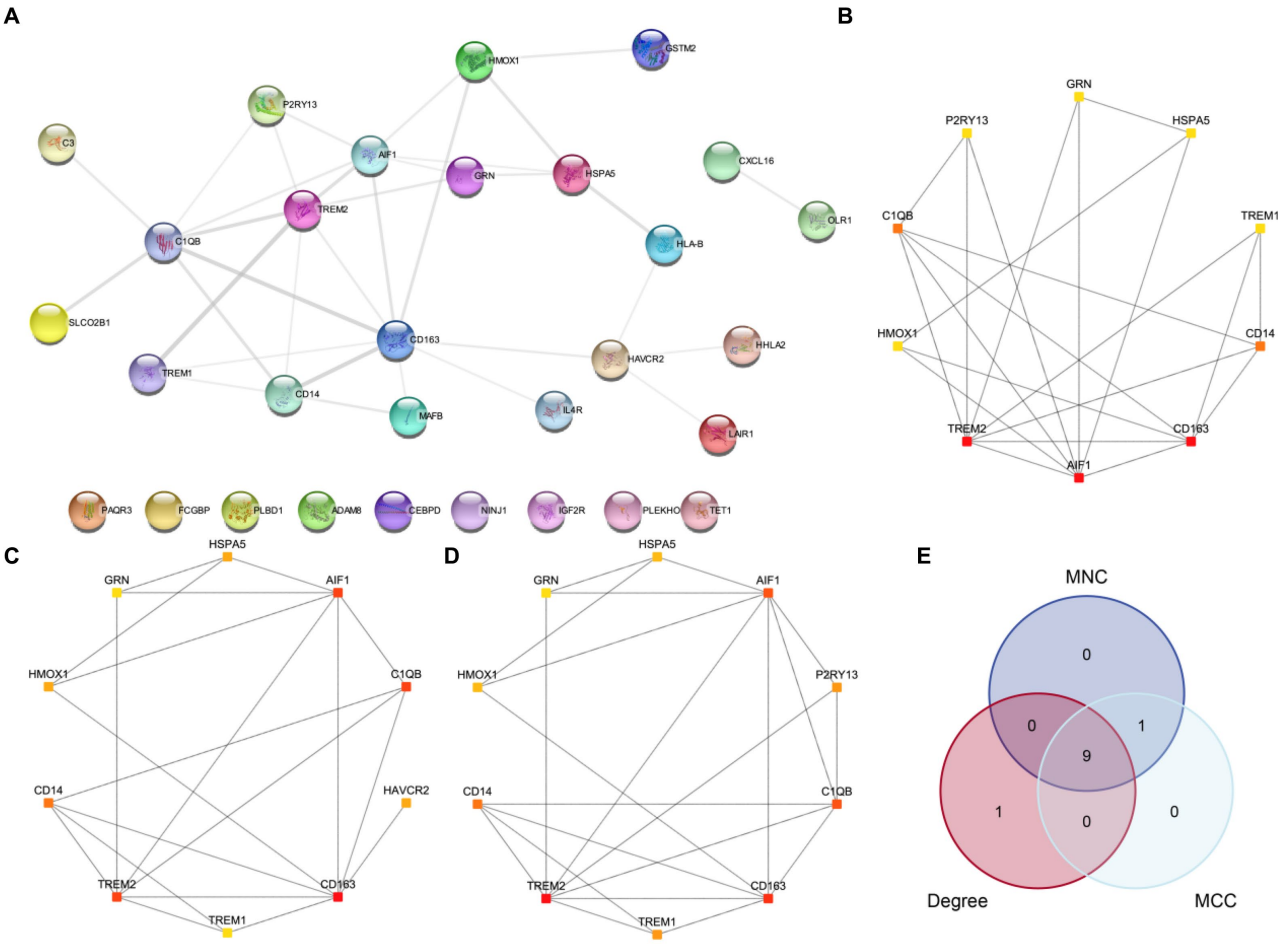
PPI network and hub genes analysis. **(A)** PPI network of MPRDEGs. **(B–D)** PPI networks of the top 10 MPRDEGs in the maximum neighborhood component algorithm **(B)**, degree algorithm **(C)**, and maximal clique centrality algorithm **(D)**. **(E)** Venn diagram of the top 10 MPRDEGs in the three algorithms. PPI Network, protein–protein interaction network; MPRDEGs, macrophage polarization-related differentially expressed genes; MNC, Maximum Neighborhood Component; MCC, Maximal Clique Centrality. The color of each square represents the score from high to low, ranging from red to yellow.

First, we conducted a PPI analysis to construct the PPI Network of 30 MPRDEGs associated with macrophage polarization using the STRING database. Cytoscape software was used to visualize the MPRDEGs exclusively (Figure 6A). The PPI Network results revealed interactions among 21 MPRDEGs, including AIF1, C1QB, C3, CD14, CD163, CXCL16, GRN, GSTM2, HAVCR2, HHLA2, HLA-B, HMOX1, HSPA5, IL4R, LAIR1, MAFB, OLR1, P2RY13, SLCO2B1, TREM1, and TREM2. Subsequently, using the Cytohubba plugin, we applied three algorithms-MNC (Figure 6B), Degree (Figure 6C), and MCC (Figure 6D) to compute scores for the MPRDEGs. Based on their scores, the 21 MPRDEGs were ranked, and the top 10 genes identified by all three algorithms were intersected to generate a Venn diagram (Figure 6E) for further analysis. Nine hub genes associated with macrophage polarization were identified: AIF1, CD163, TREM2, C1QB, CD14, GRN, HSPA5, TREM1, and HMOX1.

### 3.7 Constructing regulatory networks

**Figure 7:**
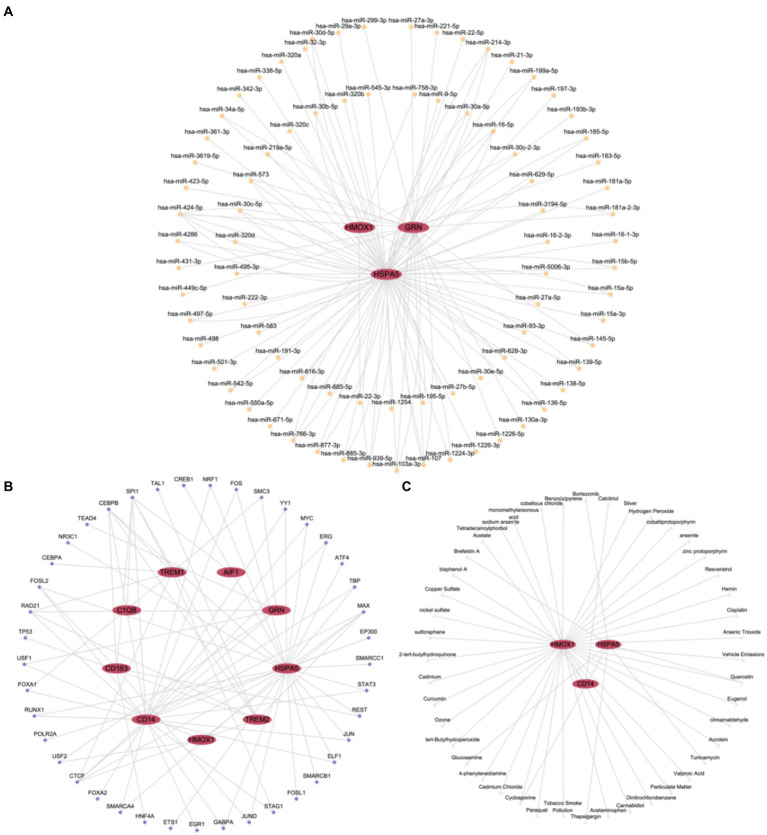
Regulatory network of hub genes. **(A)** mRNA-miRNA Regulatory Network (mRNA-miRNA regulatory network of hub genes associated with macrophage polarization). **(B)** mRNA-TF Regulatory Network (mRNA-TF regulatory network of hub genes associated with macrophage polarization). **(C)** mRNA-Drug Regulatory Network (mRNA-Drug regulatory network of hub genes associated with macrophage polarization). TF, Transcription Factor. Red ellipses represent mRNA, orange circles represent miRNA, dark blue diamond represent TFs, and light blue triangles represent drugs.

Firstly, miRNAs associated with hub genes involved in macrophage polarization were retrieved from the TarBase database. Using Cytoscape software, we constructed and visualized an mRNA-miRNA regulatory network (Figure 7A). The network included 3 hub genes (HSPA5, GRN, and HMOX) and 82 miRNAs, such as miR-424-5p, miR-107, miR103a-3p, and miR-27a-3p, among others. Further details are provided in Supplementary Table S2.

Subsequently, TFs binding to hub genes associated with macrophage polarization were identified through the ChIPBase database. Using Cytoscape software, we constructed and visualized an mRNA-TF regulatory network (Figure 7B). The network encompassed 9 hub genes (AIF1, CD163, TREM2, C1QB, CD14, GRN, HSPA5, TREM1, and HMOX1) and 41 TFs, including SPI1, CEBPB, RAD21, CTCF, and MAX. Further details are provided in Supplementary Table S3.

Finally, potential drugs or molecular compounds targeting hub genes associated with macrophage polarization were confirmed using the CTD database. Cytoscape software was utilized to construct and visualize an mRNA-Drug regulatory network (Figure 7C). The network comprised 3 hub genes (HSPA5, CD14, and HMOX) and 44 drugs or molecular compounds, such as cyclosporine, quercetin, and others. Further details are provided in Supplementary Table S4.

### 3.8 Immune infiltration analysis of polycystic ovary syndrome dataset

**Figure 8:**
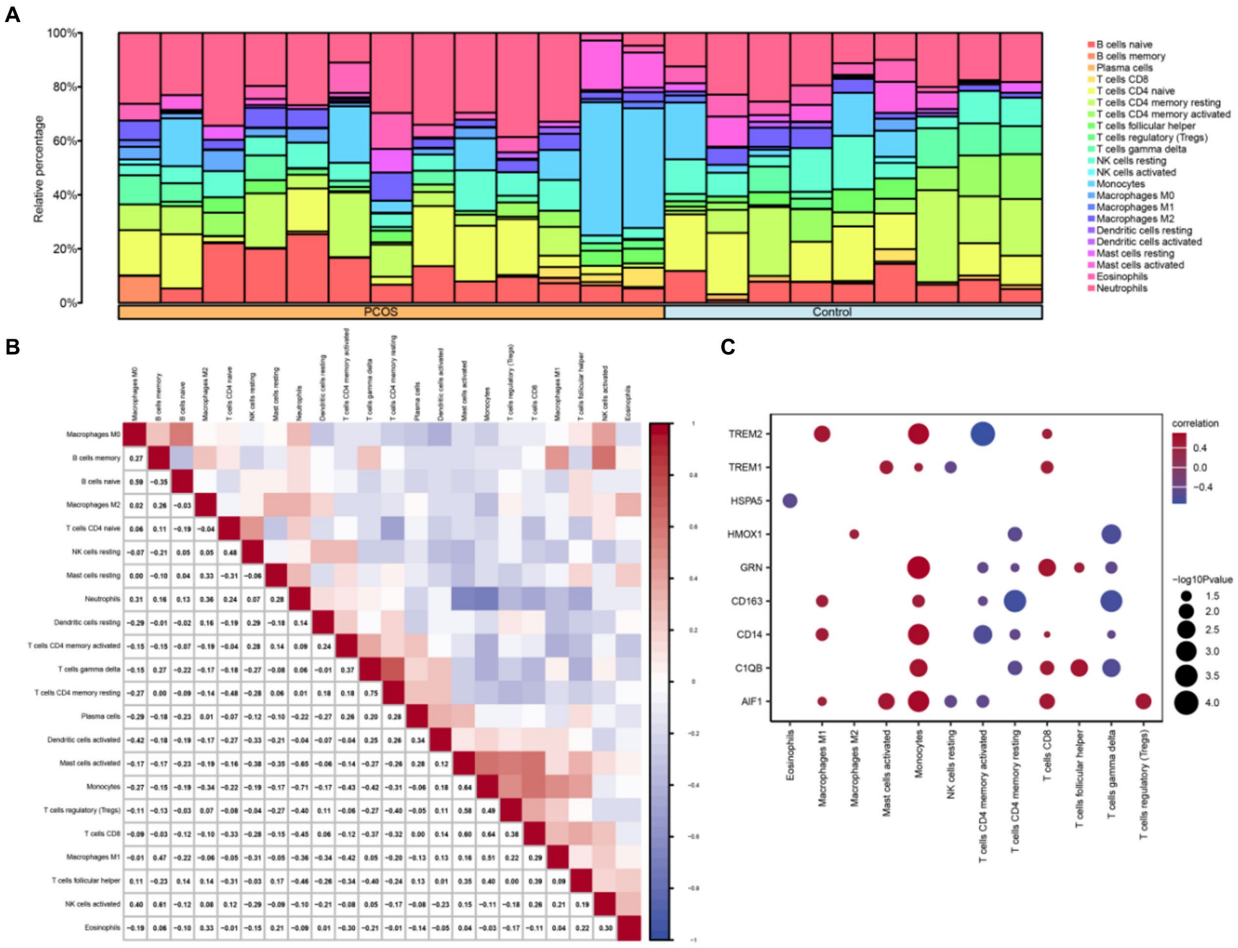
Combined datasets immune infiltration analysis by CIBERSORT algorithm. **(A)** Proportional bar chart of immune cells in the combined datasets. **(B)** Heat map of the correlation between immune cell infiltration abundance in the combined datasets. **(C)** Heat map of the correlation between hub genes related to macrophage polarization and immune cell infiltration abundance in the combined datasets. PCOS, Polycystic Ovary Syndrome. Light blue represents the control group, and orange represents the PCOS group. Blue indicates a negative correlation, and red indicates a positive correlation.

The CIBERSORT algorithm was utilized to calculate the correlation between 22 immune cell types and the PCOS and control groups based on the combined GEO dataset. Subsequently, the proportion of immune cells in the combined GEO datasets was visualized using a bar chart (Figure 8A). A correlation heatmap was then generated to illustrate the relationship in immune cell infiltration abundance (Figure 8B). The results showed that several immune cells exhibiting significant correlation: NK cells activated and B cells memory showed the strongest positive correlation. In contrast, Neutrophils and Monocytes showed the strongest negative correlation. Additionally, another correlation heatmap was generated to depict the associations between macrophage polarization-related hub genes and immune cell infiltration abundance in the PCOS dataset (Figure 8C). The results indicated that Macrophages M1, Monocytes, and CD8^+^ T cells demonstrated a strong positive correlation with macrophage polarization-related hub genes, including TREM2, GRN, CD163, CD14, C1QB, AIF1, and so on. Conversely, CD4^+^ memory-resting T cells and CD4^+^ memory-activated T cells exhibited a strong negative correlation with macrophage polarization-related hub genes.

### 3.9 Differential expression analysis of hub genes related to macrophage polarization

**Figure 9:**
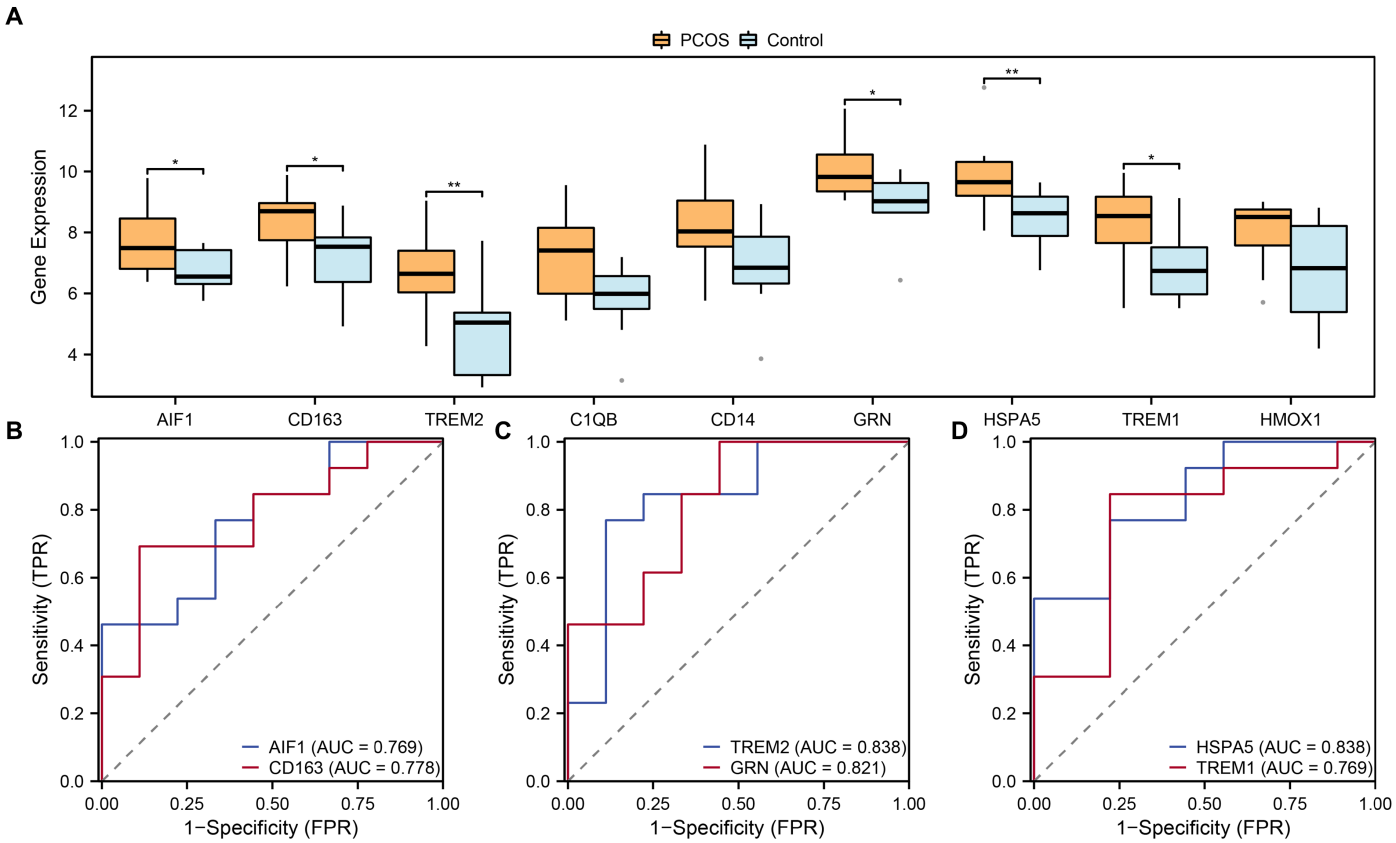
Expression difference and ROC curve analysis. **(A)** Boxplots of expression differences of hub genes related to macrophage polarization in the group comparisons in the combined datasets. **(B–D)** ROC curves of 6 hub genes related to macrophage polarization in the combined datasets. ROC, Receiver Operating Characteristic. Light blue represents the control group, and orange represents the PCOS group. “*” indicates *p* value <0.05, which is statistically significant; “**” indicates *p* value <0.01, which is highly statistically significant. AUC has a certain accuracy when it is between 0.7–0.9.

To further investigate the differential expression of hub genes associated with macrophage polarization in the integrated GEO dataset, the box plots were generated to compare the expression levels of nine hub genes between the PCOS and control groups (Figure 9A). Differential analysis of the integrated GEO dataset revealed statistical significance (*p*-value <0.05) for six hub genes related to macrophage polarization. Specifically, TREM2 and HSPA5 showed highly significant differences (*p*-value <0.01) between the PCOS and control groups. Additionally, AIF1, CD163, GRN, and TREM1 exhibited statistical significance (*p*-value <0.05) in the integrated GEO dataset comparison. Furthermore, ROC curves were plotted to assess the diagnostic potential of these six hub genes associated with macrophage polarization in the integrated GEO dataset (Figures 9B–D). The ROC curves demonstrated moderate to high accuracy (0.7 < AUC < 0.9) in distinguishing between the PCOS and control groups for AIF1, CD163, TREM2, GRN, HSPA5, and TREM1.

### 3.10 PCOS modeling and DEGs expression levels verification by qRT-PCR

**Figure 10:**
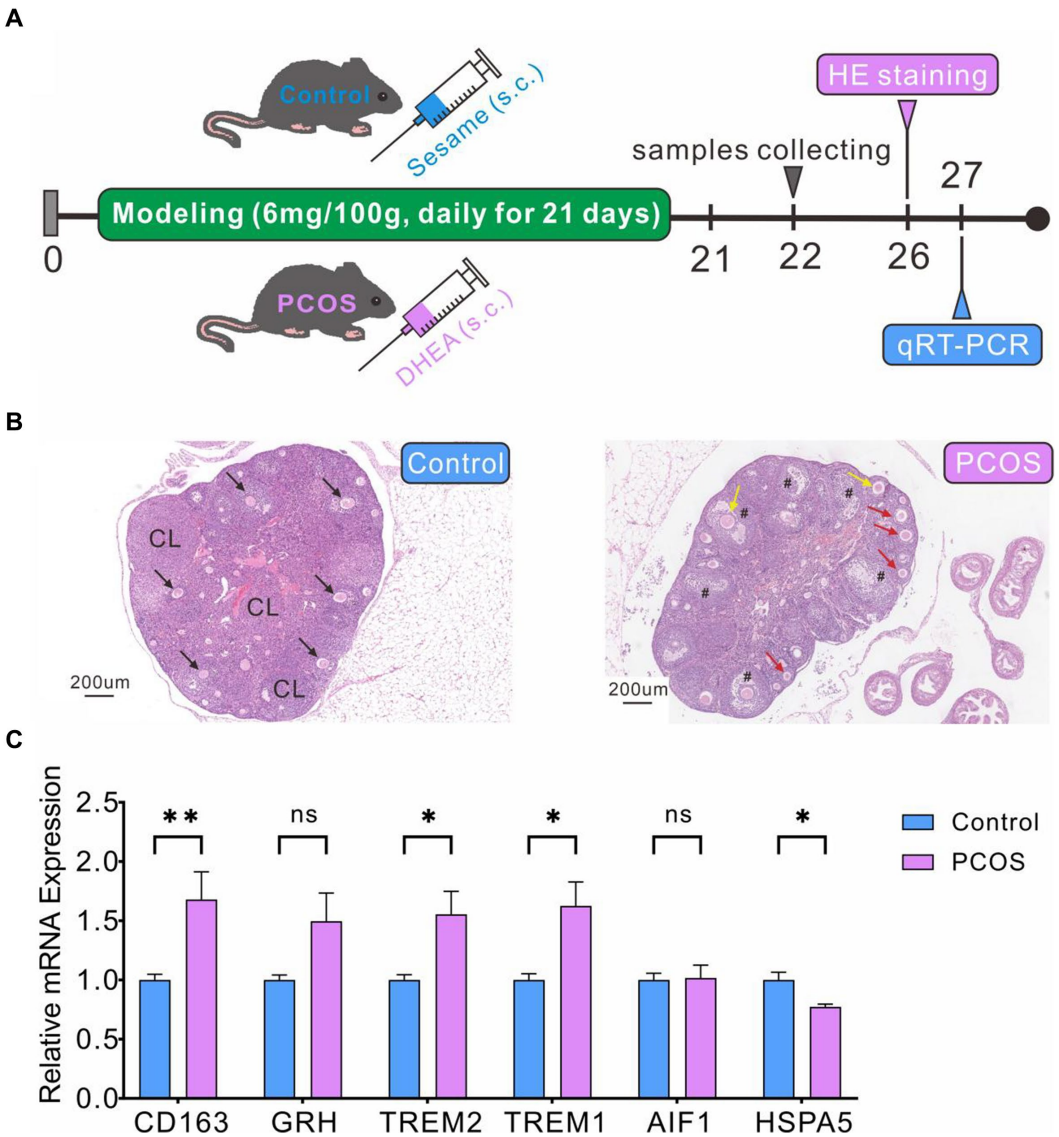
PCOS modeling and DEGs expression levels verification by qRT-PCR. **(A)** PCOS model construction and experimental flowchart. **(B)** HE staining of ovarian cross-section. Healthy follicles (black arrow), corpus luteum (CL), cystic follicles (#), immature small follicles (red arrow), atresia follicles (yellow arrow). **(C)** qRT-PCR validation of DEG levels.

We have successfully established and evaluated the PCOS model in mice (Figure 10A). HE staining revealed normal ovarian tissue in control mice, characterized by well-organized follicles and corpus luteum at various developmental stages, with orderly arranged granulosa cells (Figure 10B). In contrast, ovarian tissue from mice in the PCOS group exhibited significantly enlarged follicles, decreased granulosa cells numbers, increased immature small follicles, and absence of oocytes (Figure 10B), confirming the successful construction of PCOS model. qRT-PCR analysis indicated significantly higher expression levels of CD163, TREM1, and TREM2 genes in the PCOS group compared to the control group (Figure 10C), consistent with the database sequencing results. However, the expression levels of GRN and HSPA5 did not align with the sequencing data. GRN showed a slightly increase compared to the control group, while HSPA5 displayed a marginal decrease (Figure 10C).

## 4 Discussion

PCOS is a clinically heterogeneous syndrome characterized by various metabolic and reproductive abnormalities. Many researchers have highlighted the pivotal role of macrophage-mediated low-grade chronic inflammation in both the onset and progression of PCOS (73, 74). Specifically, understanding the mechanisms behind macrophage migration and adhesion is crucial. These processes are intricately linked to the dysregulation of ovarian function, insulin resistance, and hyperandrogenism observed in PCOS patients. By elucidating how macrophage activity influences these pathophysiological features, we can potentially uncover new therapeutic targets and diagnostic markers. Through extensive data analysis, this study identified 394 significantly up-regulated genes and 320 down-regulated genes in the PCOS group compared to the control group. Among them, 30 genes closely related to macrophage polarization were identified, predominantly enriched in molecular functions such as chemokine and cytokine production and immune response regulation. qRT-PCR experiments confirmed significantly higher expression levels of three MPRDEGs (CD163, TREM1, and TREM2) in the PCOS group. Additionally, we observed a strong positive correlation between monocytes and CD8 T cells and hub genes, while CD4 memory T cells showed a robust negative correlation with these proteins. These findings offer valuable insights into PCOS pathogenesis and potential implications for more targeted treatments and personalized management strategies in clinical practice.

Gene expression levels are crucial in regulating macrophage polarization (75). CD163 functions as a critical endocytic receptor, particularly for the haptoglobin-hemoglobin complex, marking it as an M2 type-specific marker (76, 77). In this study, CD163 expression was significantly increased and regulated by TFs such as REST and JUND. Previous studies have highlighted the enrichment of CD163^+^ macrophages in inflammation and tumor occurrence microenvironments (78), with anti-inflammatory or anti-tumor drugs targeting CD163^+^ macrophages showing promising therapeutic effects in animal models (79). Similarly, GRN is a multifunctional protein associated with inflammatory diseases, enhancing macrophage polarization towards the M2 phenotype when overexpressed. In this study, GRN was mainly involved in myoid cell activation within immune response biological processes, regulated by miRNAs such as miR-145-5p, miR-34a-5p, and miR-542-5p, as well as TFs like RAD21, CTCF, and TEAD4. However, qRT-PCR results did not show significant differences in GRN gene expression between PCOS and control mice. Additionally, TREM can serve as a specific immune target capable of activating anti-tumor immune cells (36, 80). Activation of TREM1 amplifies inflammatory responses, potentially enhancing systemic immunity. Specifically, in the context of PCOS, TREM1 activation in the wound microenvironment recruits immune cells like T cells, NK cells, DCs, and macrophages, thereby promoting the activation of anti-tumor immune cells (80). In this study, TREM1 expression was significantly up-regulated in PCOS patients and primarily regulated by transcription factors such as SPI1, CTCF, and STAG1. It is involved in scaffold protein binding and contributes to the biological process of leukocyte-mediated immunity. TREM2 has been implicated in fostering an immunosuppressive tumor microenvironment (81) and is expressed in tumor-associated macrophages, correlating negatively with cancer prognosis (82). The high expression of TREM2 in our study suggests its potential significance as a therapeutic target in PCOS. Recent research underscores TREM2’s pivotal role in various diseases, including Alzheimer’s disease, obesity, fatty liver, arterial congee, stroke, and other diseases. In addition, TREM2 expression levels appear to be regulated by TFs such as SPI1, CEBPB, and RAD21, although the specific regulatory signaling pathways remain unclear.

HSPA5 increased expression is considered an essential indicator of poor prognosis and reduced survival rate in cancer patients (83). Macrophage differentiation may affect the HSPA expression level. Previous studies have demonstrated the significant impact of macrophage differentiation into the M1 type on HSPA expression, whereas differentiation towards the M2 type does not notably affect HSPA. Notably, the expression level of HSPA5 in the PCOS group remains uncertain. Our analysis using the GEO database indicated significantly higher expression of HSPA5 in the PCOS group compared to the control group. However, qRT-PCR validation showed no significant difference between the two groups, with HSPA5 expression levels in the PCOS group even significantly lower than those in the control group. This inconsistency may be attributed to the limited sample size or our data did not directly originate from macrophages, potentially introducing bias into the conclusions. The precise expression level and functional role of HSPA5 in the PCOS group require further validation through cellular or animal experiments. Previous studies have highlighted overexpression of HSPA5 in breast, ovarian, colorectal, and other tumors, suggesting its role in regulating immune microenvironment homeostasis through apoptosis-related signaling pathways such as MAPK/ERK and PI3K/AKT (83, 84). However, in renal tumors, increased HSPA5 expression did not show a statistically significant correlation with prognostic survival and even suggested a favorable prognosis in kidney renal clear cell carcinoma (KIRC) (84, 85). The predictive utility of HSPA5 may vary with tumor type or specific subtypes. Future studies could explore targeted approaches to HSPA5 and investigate potential miRNA regulatory mechanisms and specific signaling pathways. Furthermore, HSPA5 has been predicted to have close associations with seven potential drugs or molecules (bortezomib, brefeldin, glucosamine, cyclosporine, tunicamycin, and quercetin), but their efficacy and other factors require further validation.

Although this study systematically analyzed the macrophage polarization characteristics of PCOS, several limitations should be noted. Firstly, while the sample size met statistical requirements, a larger dataset could yield more precise results. In future studies, we plan to expand the sample size and conduct an in-depth analysis of the regulatory mechanisms underlying macrophage polarization. We aim to provide more valuable insights for the pathological research and clinical management of PCOS. Secondly, this study did not analyze and validate pro-inflammatory factors related to M1 macrophage polarization (such as TNF-a, IL-6, IL-1, and IFN-γ) in PCOS samples, and factors associated with M2 macrophage polarization (such as IL-4 and IL-10) were not examined. Understanding these inflammatory factors could provide deeper insights into the immune microenvironment and disease progression of PCOS. Thirdly, This study did not use a dataset exclusively containing macrophages. However, the GSE5850 and GSE34526 datasets, which were utilized, have been shown to interact significantly with the immune system in the ovarian microenvironment and have been recognized in PCOS and inflammation research (10, 53, 54). While our study provides important insights and preliminary evidence regarding the potential role of macrophages in PCOS, the conclusions may require validation with datasets specifically focused on immune cells. Fourthly, this study did not investigate the localization of differential genes such as AIF1, CD163, and TREM2. Due to limited research resources, additional animal experiments were not feasible to validate our current findings. However, future studies will focus on exploring the expression, localization, and functions of these genes in animal models, aiming to investigate further specific signaling pathways and mechanisms regulating macrophage polarization in the progression of PCOS. Furthermore, we have not yet conducted a detailed analysis of the specific expression level differences of these miRNAs and TFs between the PCOS group and the control group. Currently, we have only analyzed miRNAs and TFs that may influence the expression of MPRDEGs through network interaction analysis. We also plan to validate our findings from mouse models in human samples in the future to ensure the clinical applicability of our conclusions. Additionally, we aim to incorporate comprehensive multi-omics data analysis and compare it with clinical samples. Our specific experimental plan includes isolating and extracting specific immune cells from the peripheral blood of PCOS patients, followed by detailed gene expression, functional analysis, and phenotypic studies of these cells. Through these approaches, we hope to uncover and validate key immune characteristics and mechanisms associated with PCOS. We also plan to validate our findings from mouse models in human samples in the future to ensure the clinical applicability of our conclusions. Additionally, we aim to incorporate comprehensive multi-omics data analysis and compare it with clinical samples. Our specific experimental plan includes isolating and extracting specific immune cells from the peripheral blood of PCOS patients, followed by detailed gene expression, functional analysis, and phenotypic studies of these cells. Through these approaches, we hope to uncover and validate key immune characteristics and mechanisms associated with PCOS.

## 5 Conclusion

In summary, our study identified 714 DEGs in the PCOS group compared to controls, including 30 MPRDEGs. Furthermore, we identified regulatory relationships where 41 TFs regulated 9 MPRDEGs, and 82 miRNAs were implicated in the regulation of 3 MPRDEGs. Additionally, 3 MPRDEGs were found to be associated with 44 drugs or molecular compounds. Using a PCOS animal model, qRT-PCR validation confirmed significant overexpression of CD163, TREM1, and TREM2 genes in the ovarian tissue of PCOS mice. This research provides insights into the polarization state and regulation of macrophages in PCOS, offering potential new drug targets for clinical prevention and treatment strategies.

## Data Availability

The original contributions presented in the study are included in the article/Supplementary material, further inquiries can be directed to the corresponding author.
